# MethylToSNP: identifying SNPs in Illumina DNA methylation array data

**DOI:** 10.1186/s13072-019-0321-6

**Published:** 2019-12-20

**Authors:** Brenna A. LaBarre, Alexander Goncearenco, Hanna M. Petrykowska, Weerachai Jaratlerdsiri, M. S. Riana Bornman, Vanessa M. Hayes, Laura Elnitski

**Affiliations:** 1 Graduate Program in Bioinformatics, Boston University, Boston, MA USA; 2 Genomic Functional Analysis Section, Translational and Functional Genomics Branch, National Human Genome Research Institute, National Institutes of Health, 49 Convent Dr., Bethesda, MD 20892 USA; 3 Laboratory for Human Comparative & Prostate Cancer Genomics, Garvan Institute of Medical Research, Darlinghurst, NSW Australia; 4 School of Health Systems and Public Health, University of Pretoria, Hatfield, Pretoria, South Africa; 5 Sydney Medical School, University of Sydney, Camperdown, NSW Australia

**Keywords:** Bisulfite sequencing, Illumina methylation array, Data analysis, Methylation probes, Single nucleotide polymorphisms (SNPs), Polymorphisms, Enhancers, CTCF sites

## Abstract

**Background:**

Current array-based methods for the measurement of DNA methylation rely on the process of sodium bisulfite conversion to differentiate between methylated and unmethylated cytosine bases in DNA. In the absence of genotype data this process can lead to ambiguity in data interpretation when a sample has polymorphisms at a methylation probe site. A common way to minimize this problem is to exclude such potentially problematic sites, with some methods removing as much as 60% of array probes from consideration before data analysis.

**Results:**

Here, we present an algorithm implemented in an R Bioconductor package, MethylToSNP, which detects a characteristic data pattern to infer sites likely to be confounded by polymorphisms. Additionally, the tool provides a stringent reliability score to allow thresholding on SNP predictions. We calibrated parameters and thresholds used by the algorithm on simulated and real methylation data sets. We illustrate findings using methylation data from YRI (Yoruba in Ibadan, Nigeria), CEPH (European descent) and KhoeSan (southern African) populations. Our polymorphism predictions made using MethylToSNP have been validated through SNP databases and bisulfite and genomic sequencing.

**Conclusions:**

The benefits of this method are threefold. First, it prevents extensive data loss by considering only SNPs specific to the individuals in the study. Second, it offers the possibility to identify new polymorphisms in samples for which there is little known about the genetic landscape. Third, it identifies variants as they exist in functional regions of a genome, such as in CTCF (transcriptional repressor) sites and enhancers, that may be common alleles or personal mutations with potential to deleteriously affect genomic regulatory activities. We demonstrate that MethylToSNP is applicable to the Illumina 450K and Illumina 850K EPIC array data and is also backwards compatible to the 27K methylation arrays. Going forward, this kind of nuanced approach can increase the amount of information derived from precious data sets by considering samples of the project individually to enable more informed decisions about data cleaning.

## Background

Interest in the role of epigenetics in human conditions, exemplified by studies in attention deficit hyperactivity disorder and autism [1], has risen exponentially from 7100 PubMed indexed articles in 2007 to over 55,400 in early 2019 [2]. This burgeoning field has identified disease-related alterations of DNA methylation ranging from type 2 diabetes mellitus to autoimmunity and cancer [3]. Outside of the disease context, there are few examples of epigenetic variations that are directly associated with phenotypic differences. In one example, differential methylation in B-lymphocytes obtained from White American, African American, and Han Chinese American individuals showed 439 CpG sites of which two-thirds were directly associated with the underlying genetic background, and one-third had no direct relation to genetic variation [4]. These findings indicate that distinct population-specific methylation patterns exist, and they result from a mixture of genetic and epigenetic causes.

Distinguishing some genetic and epigenetic phenomena can be performed by combining DNA methylation and genotyping in order to remove sequence variants that coincide with methylated positions. However, for many epigenetic studies, genotype data are unavailable—highlighting a problem plaguing the use of methylation arrays. It is difficult to distinguish between true differential methylation at a CpG site versus the presence of a SNP at that site, which can be read as differential methylation in Illumina methylation array data. This distinction is essential for properly interpreting epigenetic effects that are independent of genetic effects within distinct populations. Moreover, because SNPs create bias in methylation data, conclusions from affected epigenetic studies could be erroneous. This point is exemplified by the study of Daca-Roszak et al. [5] that showed over 68% of interrogated CpGs carried SNPs with strongly differentiating allele frequencies in inter-population comparisons.

Cytosine (C) to thymine (T) polymorphisms are the most frequent transitions occurring in the human genome; often driven by the spontaneous deamination of a methylated cytosine at CpG dinucleotides to yield thymine. The appearance of mixed pyrimidines (C and T) at a single genomic location also parallels the outcome of chemistry used to detect differential DNA methylation. In the latter case, sodium bisulfite treatment converts unmethylated Cs to Ts, whereas methylated Cs remain unchanged. Thus, a common C to T polymorphism appearing specifically in one population could be misinterpreted as differential DNA methylation between individuals. Pinpointing sites across the array where this conflation may be occurring avoids erroneously calling differential methylation.

**Fig. 1:**
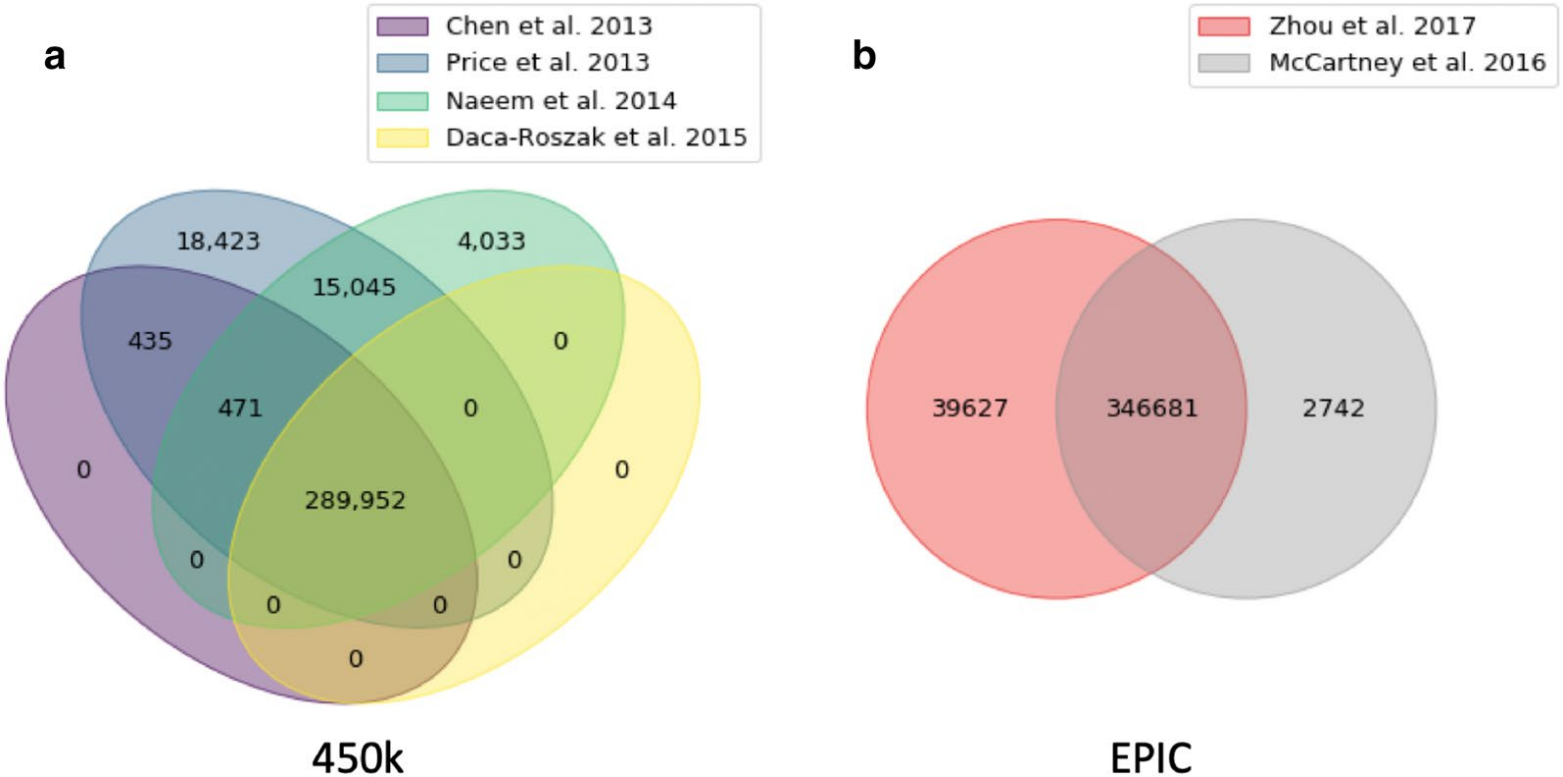
Venn diagram of known polymorphisms recommended for removal in published literature. **a** For Illumina 450K data, four publications agree on identification of 289,952 polymorphic positions [5–8], but do not agree on an additional 38,407 positions. **b** For Illumina Epic data, two publications [9, 10] agree on identification of 346,681 polymorphic positions but differ on another 42,369 positions. Data obtained via *methylcheck* python package

In general, there is broad agreement that variants can affect the performance of the arrays and influence the results, such that they should be considered when filtering data [5–10], but the approaches are not standardized (Fig. 1). One conservative approach to this problem is to remove all probe locations known to harbor human genetic variants prior to investigating methylation. Such approach could be easily implemented using *dropLociWithSNP()* function from *minfi* Bioconductor package [11, 12]. For example, a recent publication advocates removing 190,672 probes including 70,118 target CpG SNPs from the Illumina 450K methylation array data, which amounts to a loss of 39% of the available CpG sites [7]. Because many of these polymorphisms may not be present in the sequences from the studied individuals and may show methylation differences that are correlated to a disease of interest or be unique to the population being studied, this approach needlessly discards almost as much methylation data as it retains. The same problems persist in the updated 850K CpG-site Illumina EPIC methylation array, potentially including even more polymorphic sites [13]. We found 29,162 known SNPs at the target CpG site and more than 147,867 overlapping the probe body. Some papers reasonably argue that subpopulation-specific sets of SNPs would not remove as many array probes from consideration, however this approach could only be limited to studies with homogeneous ethnicity and will likely also excessively remove sites with rare variants [9]. Another approach, an alternative to filtering probes with known variants, is to mask the outlier values themselves. Such an approach is implemented in probe-wise outlier detection with *pwod()* function in wateRmelon package [14] and may be suitable for preserving the methylation data for known SNP sites with rare variants. Beyond the effect of sites harboring common alleles, positions of novel variants that are not included on genotyping arrays would create bias in DNA methylation data unless additional information from accompanying whole genome sequence data is provided. This option of having matched whole genome sequence and methylation data is unlikely to be the default situation due to prohibitively high costs.

A few published studies have identified patterns in DNA methylation data, which can be used as flags for potential polymorphisms, copy number variants, or cross-hybridizing sequences [5, 7, 8]. One method called “gap hunting” has been developed to recognize these patterns for quality control purposes [15]. The study highlighted that gap- (or whitespace) hunting in the cloud of methylation data points was a more robust approach than statistical data clustering, such as a Gaussian mixture modeling, for finding biases in methylation data created by SNPs. The method was used to flag locations in methylation array data that had characteristic clustered distributions of data points indicative of potential problems in the underlying data. The method is quite extensive and identifies up to nine categories of potential alterations, resulting in a lot of calls, furthermore its default parameters appear to be calibrated for large-scale studies.

For ease of data interpretation and application, we produced a method for detecting and removing methylation data generated specifically at C or T SNP positions in the Illumina Infinium methylation arrays and implemented it in a software package named “MethylToSNP”. Requiring only methylation array data, the method is able to identify potential SNP sites present in a sample set of interest by identifying the whitespace pattern. MethylToSNP enables researchers to avoid the overly conservative solution employed in many analyses, whereby all probe locations known to harbor human genetic variants are removed. Besides, MethylToSNP approach can be combined with existing pipelines for novel SNP discovery and postprocessing, as it does not remove the probes but rather suggests them for further consideration. We tested MethylToSNP using Illumina methylation array data containing known SNP positions, developed a confidence rating for our predictions, and validated our findings with bisulfite sequencing (for DNA methylation) and targeted Sanger sequencing or Illumina whole genome sequencing (for genotyping). To further test our method, we included methylation array data from four geographically and/or ethnically distinct populations. These included the well-characterized LCL cell line-derived samples from the Yoruba in Ibadan, Nigeria (YRI) [16] and CEU of northern and western European descent [17], each included as part of the HapMap genotyping consortium. We further applied the method to methylation array data generated from DNA extracted from whole blood for two ethnically and genetically distinct, yet geographically matched populations from southern Africa, namely the KhoeSan and Bantu populations. Finally, in these datasets, we explored the frequency of novel SNPs in areas of functional activity in the human genome, namely enhancer regions and CTCF binding sites, to further emphasize the relevance of disentangling SNP presence versus differential DNA methylation when generating biological interpretations.

## Results

### MethylToSNP overview

**Fig. 2:**
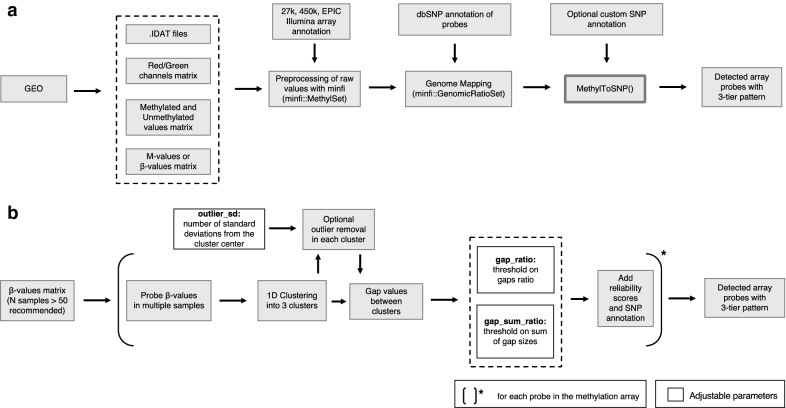
**a** MethylToSNP can be integrated in existing methylation array processing pipelines, where the source data can originate from remote data sources, such as GEO or local files. MethylToSNP requires already preprocessed data and will merge the SNP predictions with existing SNP annotations if available. This is why we recommend using it in conjunction with Bioconductor *minfi* package. **b** Schematic representation of the MethylToSNP workflow. Given a *minfi* object or a plain matrix, MethylToSNP will extract beta-values and will process one ‘cg’ probe at a time. For each probe, methylation values in different samples are clustered to find gaps between clusters with optional outlier exclusion (see “Methods”). The gaps are tested against the thresholds passed as the program’s parameters. Predicted SNPs are then reported along with the existing SNP annotation when available. Reliability scores are calculated to emphasize detection of patterns consistent with meC > T transitions

MethylToSNP predicts the location of SNPs affecting Illumina methylation array data using only a matrix of methylation values. It generates a list containing the locations of all potential SNPs in the sample set, calculates a reliability score and annotates known SNPs according to the annotation source, for instance dbSNP [18]. Following calculation of a reliability score, a user can then selectively remove SNP-affected data from the analysis. A schematic diagram of the program can be found in Fig. 2. Each of the steps of the process is described in “Methods” section (and Additional file 1).

### SNP-finding algorithm

**Fig. 3:**
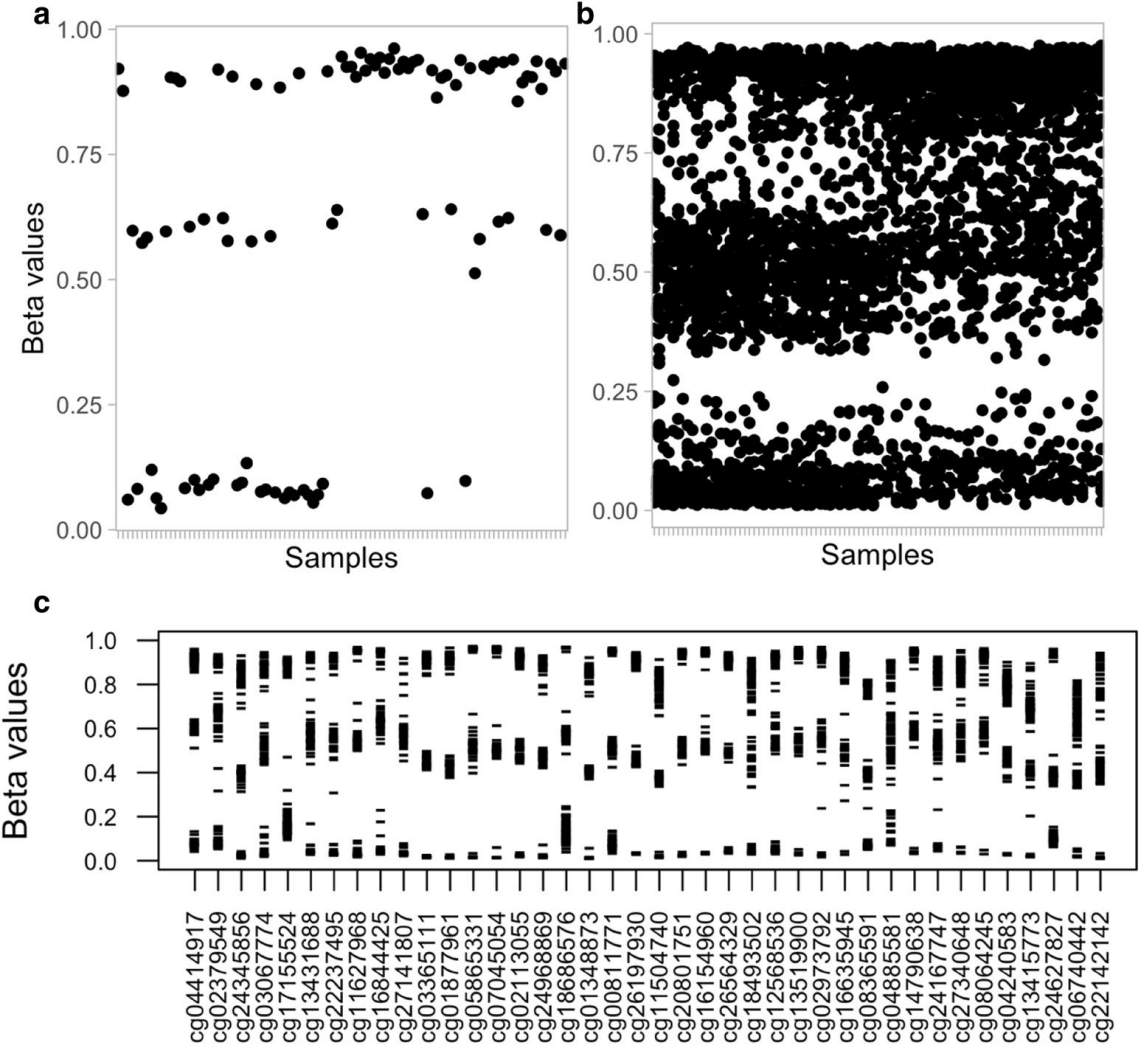
SNP presence distributes the methylation data into three tiers. All examples illustrated using 95 southern Africa samples. **a** DNA methylation beta-values at a single SNP site plotted across all samples. **b** Data from 40 randomly selected sites with SNP-like three-tier patterns plotted together across all samples to illustrate the need for variability in cutoff values used at each SNP position. **c** The same data as in (**b**) is shown separately for each array probe (i.e., genome locus). Probe-wise thresholds allow separation of data into three tiers

MethylToSNP exploits the characteristic methylation pattern found at SNP sites [5, 7, 8], to predict the presence of SNPs. A polymorphic site often returns three discrete levels of methylation: for example, full methylation would correspond to a methylated CC genotype, partial methylation would correspond to a methylated CT genotype, and the absence of methylation would correspond to a TT genotype. In these cases, β-values fall into three levels, with gaps in between, when all samples are plotted on a continuous scale of 0–1 (Fig. 3a). MethylToSNP searches for the gaps associated with this pattern. For example, a location consistent with a SNP most often has samples near the high end of the range (0.75 and higher), some samples near the middle of the *β*-value range (around 0.4–0.6), and samples with *β*-values at the low end of the range (though not necessarily zero because of background noise). This pattern is reproducible at all SNP loci, however variation in the size of the range from low to high values creates slightly different boundaries of the whitespace at each position (Fig. 3b). By contrast, *β*-values for a site without a SNP might all fall within a narrow range, or across a continuum, with no large gaps falling in between data points. Thus, when a meC > T SNP is present, the limited combination of methylation values will produce three discrete “tiers” in the data separated by data-free regions or gaps, whereas when there are no large gaps in the data field, no SNP can be predicted.

### SNP assessment in the YRI and CEU DNA methylation datasets

**Fig. 4:**
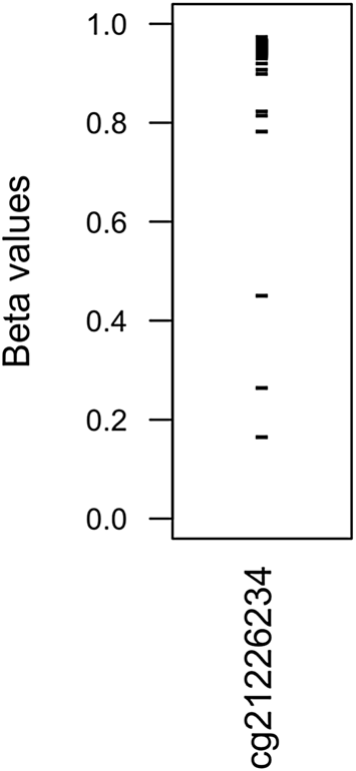
Plot of beta-values at probe site cg21226234 in Yoruba in Ibadan, Nigeria (YRI) Illumina 27K samples detected by MethylToSNP. The site received a high reliability score of 0.958. The probe at this site corresponds to a known SNP rs775651175

The YRI and CEU HapMap samples are well studied, limiting the expectation of additional novel polymorphism detection and providing a reference dataset for testing the software tool. We tested results at 24,000 methylation probes in 77 YRI samples and 90 CEU samples. MethylToSNP flagged seven sites in the YRI data that we could easily validate. Moreover, the program also reported reliability scores at each site above the threshold of 0.5 (see “Methods”). Two of the seven sites were present in dbSNP version 146 (i.e., methylation probes cg21505334 and cg21226234) with heterozygous alleles reported for position cg21226234 (illustrated in Fig. 4) in 1000 Genomes Browser sequence data. Two other sites dated back to the previous database release, dbSNP version 142 (cg08261841 and cg09953122). The three remaining sites (cg02119982, cg16757724, and cg22484980), were adjacent to known SNP-containing positions, which would affect hybridization of the probes in the methylation assay. In addition to the validated sites, we identified six sites in YRI and 31 in CEU with reliability scores above 0.50, which would be strong candidates as novel SNPs (Table 1).

**Table 1:** Variants predicted by applying MethylToSNP to the YRI and CEU HapMap methylation datasets

Set	SNP predictions in dataset (#)	Overlap with dbSNP 142 (#)	Overlap with dbSNP 146 (#)	Potential novel SNPs^a^ (#)	Total array sites removed by dbSNP 142 filtering^b,c^ (#)	Total array sites removed by dbSNP 146 filtering^b,c^ (#)	Median reliability scores of predicted SNPs	# novel SNPs with reliability scores ≥ 0.5
YRI	37	3	9	28	3563	5677	0.01875	6
CEU	283	37	57	226	3329	5629	0.011	31

Default threshold values used

^a^After filtering for known SNPs

^b^Direct overlap of C in CpG position

^c^After removing MethylToSNP

In total, the program predicted 37 and 283 potential SNP positions (Table 1). The known SNP positions intersected 9 and 57 positions that carried SNPs in these datasets (using dbSNP146). The remaining 28 positions in YRI and 226 sites in CEU also carried distinctive patterns that resembled SNPs. We examined these positions using MethylToSNP reliability scores (see “Methods”) and found that, except for the six sites in YRI and 31 in CEU, the majority scored below the threshold of 0.50, with a median score of just 0.02 for the YRI predictions and 0.01 for the CEU predictions, suggesting that few of these sites would be viable meC > T SNP candidates.

To confirm that predictions with low reliability scores did not represent SNPs, we selected three of the 28 YRI results, which had three-tiered methylation patterns in 15 of the 77 YRI samples. Reliability scores for the three examples were low (0.042, 0.034, and 0.070), and the majority of the data points occupied the lower portion of the beta-value range (representing unmethylated Cs). Through bisulfite sequencing we confirmed the presence of DNA methylation creating the three-tiered methylation levels, whereas targeted Sanger sequencing showed no polymorphisms (Table 2). Furthermore, only one sample had a SNP within 50 bp of the CpG of interest, whereas anything greater than 10 bp is reported unlikely to influence methylation levels [7, 8]. These results show that individual loci can display heterogeneous levels of DNA methylation that mimic a heterogeneous nucleotide pattern in the Illumina methylation data, which can be filtered from potential meC > T SNP sites using the MethylToSNP reliability score. Notably, probe cg23886551 is associated with a gene reported to be imprinted (*TMEM121* [19]), whereas cg18335068 is associated with a gene that has at least one report of monoallelic expression (*ZNF677* [20]).

**Table 2:** Sequencing results of three sites in the YRI predictions

Probe of interest (genomic position)	MethylToSNP reliability score from 70 samples	Example genome	Average methylation level from array data	Predicted genotype (bisulfite-treated)	Average methylation level from bisulfite sequencing	Sequenced genotype
**cg06192753** chr19:13068298	0.042	NA19131	0.703	C/C	0.732	C/C
		NA18506	0.440	C/T	0.435	C/C
		NA18912	0.127	T/T	0.001	C/C
**cg23886551** chr14:105992620	0.034	NA19172	0.830	C/C	0.913	C/C
		NA18503	0.405	C/T	0.446	C/C
		NA19222	0.113	T/T	0.020	C/C
**cg18335068** chr19:53757911	0.070	NA18861	0.752	C/C	0.801	C/C
		NA19161	0.510	C/T	0.500	C/C
		NA18505	0.228	T/T	0.181	C/C

### Assessing novel polymorphisms in lesser characterized populations

MethylToSNP (${\text{gap}}\_{\text{ratio}} = 0.75, {\text{gap}}\_{\text{sum}}\_{\text{ratio}} = 0.5,$ no outlier removal) was applied to methylation array data generated from the epigenomes of individuals self-identifying ethno-linguistically as KhoeSan or Bantu-speaking southern Africans, providing comparative populations. From 473,767 methylation probe sites on the Illumina 450K arrays, we identified 2296 potential meC > T SNPs. Known SNPs in dbSNP146 accounted for 1402 of these sites (Table 3), giving a minimum estimated true positive rate of > 61%. The remaining 894 sites represent potential novel variants. Their median reliability score was 0.979, from which 827 sites had reliability scores ≥ 0.5. This result suggests a true positive rate of 2229/2296 or 97%, which is comparable to our sensitivity from simulated datasets (see “Methods”). Compared to the conventional approach of removing all ~ 144,000 known SNP sites overlapping the Illumina methylation array data [7], we note that removal of only 2296 potential SNPs prevents the loss of vast amount of data points from the southern African epigenomic data. This finding emphasizes the need to filter data carefully for the presence of SNPs, but not to use overly conservative approaches that indiscriminately remove all possible SNP positions, especially when including a population from Africa with known elevated levels of genetic diversity. Notably, many of these variants are newly identified, with 153 added since dbSNP142.

**Table 3:** MethylToSNP predictions in the southern African data set

Description	Probes tested (#)	# SNP predictions in dataset	Overlap with dbSNP 142 (#)	Overlap with dbSNP 146 (#)	Potential novel SNPs (#)	Sites lost by filtering all dbSNP 142 positions (#)^b^	Sites lost by filtering all dbSNP 146 positions (#)^b^	Median reliability scores of predicted SNPs	# novel SNPs with reliability scores ≥ 0.5
All probe sites	473,767	2296	1249	1402	894	101,558	144,569	0.979	827
Differential methylation^c^	12,613	23	19	19	4	2143	3081	0.395	2
Top 5% differential methylation^a,c^	400	1	0	0	1	0	48	0.779	1

^a^After filtering for known SNPs

^b^Direct overlap of C in CpG position

^c^Manuscript under revision

### Differentially methylated sites between KhoeSan and Bantu southern Africans, filtered for SNPs

For a comparison of differential methylation between the KhoeSan and Bantu southern Africans (manuscript under revision) we used MethylToSNP to identify and remove putative SNP-containing probes from the analysis. Prior to complete SNP removal, we further investigated one SNP prediction, at a position of a known SNP at Illumina methylation probe cg00117311. We predicted a SNP with a reliability score of 0.963, where two KhoeSan individuals showed 50% methylation (beta-value), suggestive of a heterozygous allele pattern (Table 4). Four available genomic sequences from KhoeSan individuals confirmed our predictions of a polymorphism, by showing that two individuals carry a C to T SNP [21], whereas the two others have only the reference allele (raw data available from Penn State Genome Browser [21, 22]). The latter two individuals showed ~ 95% methylation, indicative of a homozygous allele, from the methylation data.

**Table 4:** Sequence data verifies the presence of a SNP identified using MethylToSNP

Subject	Methylation beta-value at cg00117311	Putative alleles	Allele frequencies at rs78210031
KB1	0.458	CT	0.5 C/0.5 T
TK1	0.542	CT	0.5 C/0.5 T
NB1	0.954	CC	C
MD8	0.949	CC	C

Sequence data were obtained from the Penn State Genome Browser for four KhoeSan samples

We examined four additional probe sites, which indicated novel, but unproven SNP positions. We confirmed the presence of both common and rare variants by assessing several genomic sequences within 10 bp of the original CpG positions (Table 5). One site, cg10633981, represented the single unrecorded polymorphic site in our top 5% of differential methylation data. It has a reliability score of 0.779 further suggesting suitability as a novel polymorphism. The polymorphism does not occur in the four sequenced KhoeSan genomes which are available, and thus we examined additional sequence data to check this position. However, the prediction was not confirmed at cg10633981 due to a lack of sequence data for the individual carrying the predicted variant.

**Table 5:** Sequence data verifies the presence of SNPs identified using MethylToSNP

cg ID^a^	Chromosome	CpG position (hg38)	SNP coordinate within 10 bp	rs ID	Information
cg00786635	chr1	25,267,710–25,267,711	25,267,707	rs145726224	Common in African populations including southern Africans
			25,267,714	N/A	Rare, found in one southern African
cg07482220	chr6	32,178,742–32,178,743	32,178,737	rs112124640	Rare, identified in 3 southern Africans
cg18976974	chr8	102,978,096–102,978,097	102,978,097	N/A	Rare, only in one genome
cg10633981	chr11	16,758,221–16,758,222	N/A	N/A	No WGS data for genome of interest

^a^cg ID identifier from Illumina array annotations

### Distinguishing differential DNA methylation or SNPs in enhancers and CTCF sites

To distinguish whether methylation was affecting enhancer regions, we identified a list of 102,559 CpG positions on the Illumina 450K array overlapping annotated enhancers in the human genome. We narrowed the list to 1235 enhancer positions that overlap 12,613 differentially methylated probe positions between the KhoeSan versus Bantu southern Africans (Mann–Whitney *U* test with a Bonferroni correction). Of these, 892/1235 or 72% overlapped positions known to contain SNPs in some population (using dbSNP146). By contrast, MethylToSNP predicted only six of the 1235 sites as carrying SNPs (all of which have reliability scores over 0.5), all recorded in the set of 892 known SNP positions, suggesting that the majority of these 892 positions are differentially methylated enhancers in the KhoeSan versus Bantu comparison, and not polymorphic sites in these individuals. Again, these data suggest that removing all known SNP sites from a methylation dataset could unnecessarily eliminate potentially informative data. We also applied MethylToSNP to the full set of 102,559 enhancer CpGs represented in methylation data from our KhoeSan and Bantu samples. MethylToSNP predicted 685 high confidence SNP positions (where reliability scores ≥ 0.5), of which 327 sites overlap with known SNP sites in the human genome and occur in these samples. This approach also identifies 358 positions that could be novel SNPs that fall within enhancer positions in the KhoeSan or Bantu southern African data, although not identified as significantly differentially methylated positions.

We also examined predicted SNPs versus DNA methylation at CTCF sites in the southern African, CEU, and YRI datasets. CTCF sites coincided with 79,856 CpG probe positions on the 450K array. Using MethylToSNP we narrowed the list to 3279 differentially methylated CTCF sites (Mann–Whitney *U* test with a Bonferroni correction) between the KhoeSan and Bantu groups and “confirmed that” they did not contain SNPs. These results implicate methylation differences rather than polymorphisms at the CTCF sites. When we examined CTCF sites without differential methylation, 101 were predicted by MethylToSNP to contain SNPs in the southern African dataset (reliability scores ≥ 0.5). By contrast, only 18 of the 79,856 CpG containing CTCF sites showed SNP whitespace patterns in the data from YRI individuals. These were ruled out as SNPs because they carried a median reliability score of 0.006, with none reaching a score threshold of 0.5. Thus, the patterns are implicated in variable methylation at these CTCF sites rather than polymorphisms. Likewise, in CEU individuals 61 CTCF positions resembled SNP patterns, but collectively had a median reliability score of 0.017, carrying no individual reliability scores > 0.5. Hence, the presence of novel SNPs and differential methylation in southern African data has the potential for a functionally relevant impact in genome biology, by interfering with CTCF binding, through different mechanisms. By contrast, in YRI and CEU samples, the CTCF sites appear to carry variable methylation but not polymorphisms. Thus, the MethylToSNP analysis approach can inform SNP content in functional locations, a feature that provides value-added for samples that lack genotype information.

### Comparison to gap hunting analysis

Another recently proposed method called “gap hunting” also uses array data to identify CpG sites at which different methylation levels are present, by looking for gaps in data points [15]. The method can identify up to nine tiers of data, which result from different SNP signals, indels, and copy number variants. However, the authors do not make specific claims about what each type of pattern might represent, only cautioning that flagged sites should be considered separately in the methylation analysis. In order to compare the results to MethylToSNP, we focused on gap pattern labeled “3-groups” (consistent with two whitespace gaps). In a qualitative comparison of the tools using our datasets, we found that the list of flagged sites from each tool is not identical, and MethylToSNP’s list of problematic sites is more conservative (Table 6) when using default software parameters. For example, the gap hunting approach flagged 8486 positions compared to 381 predicted by MethylToSNP. There are also cases where MethylToSNP identifies sites that do not fall neatly into the gap hunting category called 3-groups. The differences likely arise because of the fact that MethylToSNP focuses on finding one high confidence pattern (i.e., meC > T SNPs, while enforcing an extreme range of beta-values), whereas gap hunting generally looks for data patterns with a wide range of gap-space patterns, which may represent many different types of genomic events, including annotated SNPs, indels, microsatellites, or multi-nucleotide polymorphisms. These groups can cover small or large ranges of beta-values.

**Table 6:** Comparison of MethylToSNP calls and gap hunting calls

Data set	MethylToSNP predictions	Gap hunting 3-groups predictions	% Overlap in MethylToSNP with 3-groups predictions (%)^a^	% Overlap in MethylToSNP including all gap hunting groups (%)^b^
27K YRI and CEU	371	8486	47	100
27K CEU	283	8416	44	100
27K YRI	37	3409	73	97

*YRI* Yoruba in Ibadan, Nigeria population, *CEU* CEU HapMap

^a^Feature results found in gap hunting 3-group results

^b^Feature results found in any of 9 gap hunting groups

## Discussion

Here we introduce the approach of MethylToSNP, which assesses methylation array data for the presence of polymorphic sites, which confound methylation analysis. Using a set of differentially methylated sites known to contain SNPs, we showed a true positive rate of at least 96% (based on known CpG position SNPs). Additionally, we identified several sites that may harbor previously uncharacterized SNPs or rare variants in two genetically understudied populations from southern Africa, as well as the more well characterized data from two well-characterized HapMap populations. We also find evidence of sites with potential for parent-of-origin imprinting. In short, MethylToSNP allows researchers to gain confidence in DNA methylation analysis results, while avoiding the twin problems of (a) confounding meC > T SNPs at target CpG sites and (b) needlessly eliminating large amounts of data by removing every methylation probe that has ever been associated with a polymorphic position in the genome, whether the SNP is present in the queried genomes or not.

MethylToSNP identified putative novel variants in YRI, CEU, and southern African genomes by using methylation data. Somewhat surprisingly, novel variants were predicted in the well-studied YRI and CEU samples, some of which were validated in a recent update to the dbSNP repository (dbSNP146). In other cases, although the tool initially identified potential SNPs, our reliability score correctly predicted that the sites harbored differential methylation and not polymorphisms. We verified this conclusion with methylation-based and nucleotide sequencing approaches. Other benefits of the approach include identifying differential methylation and variant occurrences in functional elements such as CTCF sites and enhancer regions when those CpGs appear on the methylation arrays.

Limitations of the study include situations where we predict novel variants, but have no genomic material to confirm their presence. This occurs in samples with minimal DNA collection amounts, such as the southern African samples. We also caution that MethylToSNP will miss predictions of a SNP location if the C is always unmethylated, which will never appear as differential methylation. In this case, there is no way to distinguish a bisulite converted C (becoming a T) “from” a polymorphic T in the genome. Therefore, we expect MethylToSNP will be less likely to detect polymorphisms in CpG island regions because they tend to be less methylated than non-CpG island locations (known as open sea locations). The majority of the locations found in the southern African data are in fact in open sea regions (1411 of 2296 calls across the whole array data set). In addition, other SNP-associated patterns besides the three tiers that MethylToSNP detects, will not be reported with this tool. In accordance with other published works [5–10], there are genomic variants like indels and copy number polymorphisms that we will not identify. We suggest complementary approaches for such inquiries. Another limitation that we have addressed in “Methods” is the minimal number of samples required for reliable detection of SNP positions. Our benchmarks indicate that the recommended minimal number of samples is 50.

As the breadth and depth of population-level epigenomic projects increase, having an optimal approach for addressing the effect of variants on methylation data will become more important. Until such a time as genotyping and methylation analyses can be performed in concert, as future sequencing technologies portend [23], MethylToSNP represents a viable approach for retaining as much methylation array data as possible while eliminating sites associated with SNPs in a given population. Moreover, MethylToSNP can be used to identify novel SNPs in the vast collections of methylation data that already exist—including more than 800 projects using the Illumina Infinium 450K array, more than 160 projects using Illumina Infinium EPIC, and more than 320 projects using the Illumina 27 K array in GEO, which include thousands of samples. Additionally, MethylToSNP has the potential to reanalyze the full spectrum of these data with the more moderate approach of only removing SNPs that are detected in the individual genomes being examined. Finally, this method could be extended to include other potential SNP signatures as outlined by others [6, 15] to create an even more comprehensive method.

## Conclusions

We describe an approach, MethylToSNP, and predicted new SNPs residing in genomes for which DNA methylation data were collected. The identification of the SNP positions enables the user to remove data points from the methylation analysis that are CpGs confounded by SNPs, without removing all potential genomic positions recorded to harbor a SNP in any given population. We used the tool to illustrate the detection of SNPs or differential methylation in functional regions of the genome, such as enhancers and CTCF binding sites, for which either event could have biological impact, but with distinctive underlying regulatory mechanisms.

## Methods

### MethylToSNP overview

MethylToSNP predicts the location of SNPs affecting Illumina methylation array data. The program takes methylation array data for multiple samples (at least 50 samples recommended) as an input and generates a list containing the locations of all potential SNPs in the data set. After a three-tier pattern is identified, postprocessing can be performed with annotation of probes and SNPs (mainly based on dbSNP database [18]) available in Bioconductor. For instance, sites can be filtered according to their location within the probe or directly on the CpG site or probes could be stratified as known or potentially novel SNPs. MethylToSNP was created in the R programming language [24] as part of the R Bioconductor ecosystem. The typical workflow is illustrated in Fig. 2a, where the input data may be originating from a remote (e.g., GEO) or local source in the format of raw array signal or already preprocessed methylation values. MethylToSNP will accept user input in the format of beta-values or, preferably, in the format generated by the BioConductor package *minfi*. The latter is preferred because the *minfi* data format incorporates genomic mapping and SNP annotation of array probes.

MethylSNP R package is available via GitHub https://github.com/elnitskilab/MethylToSNP.

### Three-tier pattern with gaps

To detect a position where methylation values are affected by a SNP either at the target CpG or its neighboring position [5], the methylation data has to be discretely separated by two gaps of similar width, where these gaps contribute to the majority of the total data range (Fig. 3). The algorithm clusters methylation data into three clusters, favoring clusters located farther away from each other, and optionally disregards outliers, and then evaluates the gaps between clusters.

Because clustering of beta-values is a one-dimensional problem, and the number of clusters is low, it can be solved optimally with dynamic programming *k*-means implementation rather than with randomly initialized *k*-means algorithm that is not guaranteed to converge to an optimum. We relied on an implementation in R package Ckmeans.1d.dp [25].

Larger clusters will naturally have higher weight than clusters only consisting of a few data points. If untreated, this problem could lead to detection of multiple clusters in highly populated data ranges (e.g., beta-values 0.7–0.9). However, in fact, we are interested in detecting large and small clusters across the whole span of beta-values. Therefore, we used weights inversely proportional to the number of samples, i.e., inverse quantile density. For quantile $q$ and the number of samples $N_{q}$ clustering weights were calculated as follows:

$$ w_{q} = \frac{1}{{N_{q} }}. $$

Additional file 1: Figure S3 illustrates the effect of inverse quantile weighting on the YRI beta-values at cg21226234 probe.

The gap between clusters can be defined as the difference in methylation levels between the bordering samples in each cluster, for instance gap between clusters $A$ and $B$, where a and b are methylation values of bordering samples, such that $\forall a \in A > \forall b \in B$:

$$ d_{A - B} = \mathop {\hbox{min} }\limits_{a} A - \mathop {\hbox{max} }\limits_{b} B. $$

After gaps are identified, a subsequent method is used to assess the size of the data-free gaps at each methylation site using two adjustable cutoffs: the ${\text{gap}}]_{\text{sum}}\_{\text{ratio}}$ value and the ${\text{gap}}\_{\text{ratio}}$ value. The ${\text{gap}}\_{\text{sum}}\_{\text{ratio}}$ approach evaluates the total gap size by summing the size of the gaps and testing whether it represents a majority of the *β*-value range. By contrast, the ${\text{gap}}\_{\text{ratio}}$ approach compares sizes among the two largest gap regions and tests whether their relative sizes are roughly equivalent. To pass this threshold, the size of the smaller gap must be at least a certain percentage of the larger gap. For example, if the ${\text{gap}}\_{\text{ratio}}$ is set to 0.75, and the larger gap spans 0.3 *β*-value, the smaller gap must span at least 0.225 *β*-value. For the algorithm to identify possible SNP locations, thresholds for both the ${\text{gap}}\_{\text{sum}}\_{\text{ratio}}$ and the ${\text{gap}}\_{\text{ratio}}$ must be met. This method allows for variability in the methylation values, while still covering a majority of the whitespace, caused by compression of the *β*-value range away from upper or lower boundaries of 1.0 and 0, respectively. Additionally, we benefit by avoiding use of a fixed cutoff to separate methylation values into levels, such as thirds or quadrants. As shown in Fig. 3b, it is typically impossible to define fixed cutoffs that would work for all probes.

Considering the two gaps between three clusters ${\text{H}}, {\text{M}}, {\text{L}}$—“high”, “mid” and “low”: $d_{{{\text{H}} - {\text{M}}}}$ and $d_{{{\text{M}} - {\text{L}}}}$, the threshold parameters ${\text{gap}}\_{\text{ratio}}$ and ${\text{gap}}\_{\text{sum}}\_{\text{ratio}}$ for the algorithm are defined as:

$$ \frac{{{ \hbox{min} }(d_{{{\text{H}} - {\text{M}}}} ,d_{{{\text{M}} - {\text{L}}}} )}}{{{ \hbox{max} }\left( {d_{{{\text{H}} - {\text{M}}}} ,d_{{{\text{M}} - {\text{L}}}} } \right)}} \ge {\text{gap}}\_{\text{ratio,}} $$

and

$$ \frac{{d_{{{\text{H}} - {\text{M}}}} + d_{{{\text{M}} - {\text{L}}}} }}{{\hbox{max} \left( {{\text{H}} \cup {\text{M}} \cup {\text{L}}} \right) - { \hbox{min} }\left( {{\text{H}} \cup {\text{M}} \cup {\text{L}}} \right)}} \ge {\text{gap}}_{\text{sum}}\_{\text{ratio,}} $$

where the denominator is the total range of beta-values across all three clusters.

### Calibrating default MethylToSNP parameters

First, two simulated data sets were created to test the ability of MethylToSNP to identify SNP-associated methylation patterns when different proportions of samples (i.e., data points) were present at each tier level. The datasets included 95 samples each, to mimic the size of the southern African data set, and circa 10,000 probe loci. In both data sets, half of the probes corresponded to non-SNPs that were drawn from the actual southern African data. The second half of the probes represented SNPs and were generated in a different way depending on the set: in the “set-frequency” dataset unequal distribution of methylation values across the tiers was generated, corresponding to low minor allele frequency (MAF) scenario, whereas in the “uniform-frequency” dataset the methylation values were distributed equally across the tiers, simulating the high MAF scenario, characteristic for common SNPs. The procedure is described in more detail in Additional file 1, along with the set frequencies and the code to reproduce the data. We used these simulated datasets to calibrate the default values of MethylToSNP parameters: the ${\text{gap}}\_{\text{sum}}\_{\text{ratio}}$ and the ${\text{gap}}\_{\text{ratio}}$. To choose the defaults (${\text{gap}}\_{\text{sum}}\_{\text{ratio}} = 0.50$, ${\text{gap}}\_{\text{ratio}} = 0.75$), the parameters were altered in 0.05 increments (see Additional file 1: Figure S1). With these parameter thresholds, the benchmark returned 97% true positive rate on “set-frequency” dataset. The uniformly simulated data set returned 100% true positive rate. In all cases there were no false positives.

However, the simulated SNP probes had a clear separation between the tiers of methylation values, thus making it difficult to assess the performance in case of presence of noise or other confounding factors.

Therefore, we created a second benchmark to assess false negative rates using the 59 control SNP probes placed by array designers on the Illumina EPIC arrays. Also to demonstrate the use of the approach on the Illumina EPIC we tested 152 pediatric samples from GEO GSE137682 dataset, where MethylToSNP with default parameters identified 41 out of 59 positions for 27% false negative rate (Additional file 1: Figure S2). However, we note that 18 control SNPs were A > G transitions or located further away than 2 bp from the CG position on the array, which we would not intend to find with our first pass approach. The remaining C > T and T > C (14 and 15, respectively) and G > A (12 total) were correctly identified.

The benchmark figures (Additional file 1: Figure S2A, B) showed that the ${\text{gap}}_{\text{ratio}}$ value can be lowered from 0.75 to 0.50 to retrieve more hits. However, the major hindrance to detection of gap patterns is the presence of noise or otherwise confounded measurements with methylation values between the tiers. In order to make the method insensitive to such measurements we implemented an outlier detection option ${\text{outlier}}_{\text{sd}}$ that is the measurement of the allowed within-cluster variance (in standard deviations). For instance, a sample with beta-value $\beta$ is an outlier in the cluster ${\text{C}}$ with the cluster center $\mu_{\text{C}}$ and variance $\sigma_{\text{C}}^{2}$ if the following threshold is not satisfied:

$$ \frac{{\beta - \mu_{\text{C}} }}{{\sigma_{\text{C}} }} \le {\text{outlier}}_{\text{sd}} . $$

In case when the outlier filtering option is enabled, any beta-value that belongs to a cluster but does not match the threshold would be excluded from the calculation of gaps between clusters. An additional benchmark run with outlier filtering enabled (Additional file 1: Figure S2D, E) showed that this option completely rescued retrieval, with zero false negatives, even in complicated cases.

We encourage users to use our benchmarks as a guidance for changing the default parameter values. Alternatively, users can recalibrate the thresholds using their own predefined control probes, for instance known SNPs, or simulated datasets.

### Size of the dataset required for the analysis

The algorithm relies on identification of three clusters, therefore the absolute minimum number of samples required for the analysis is three. However, the SNP patterns may only be detectable with larger datasets, particularly for the rare alleles. While the low MAF SNPs will set the upper detection boundary, we wanted to calibrate the lower boundary, i.e., the minimal recommended number of samples for the analysis based on common SNPs with MAF close to 0.50. We used the false negative detection rate of SNP control probes for the 152 pediatric samples from GEO GSE137682 dataset as a benchmark (Additional file 1: Figure S2C). The plot shows how many true SNP probes are retrieved in case of subsampling without replacement from 5 to 150 data points out of 152, with a step of 5, with 30 replicates. The saturation is reached at about 50 samples (i.e., data points). Removal of outliers improves the overall retrieval; however, it does not affect the lowest boundary on the number of samples required to find the three-tier methylation pattern (Additional file 1: Figure S2F). Based on this benchmark we, therefore, recommend that the size of the datasets analyzed with MethylToSNP should not be smaller than 50 samples. The program will run with 3 or more samples but will print a warning message if supplied data is insufficient for reliable detection of SNPs.

### SNP-reliability score and thresholds

MethylToSNP quantitatively assesses how close the observed methylation pattern resembles the expected meC > T SNP by providing a reliability score. In general, the majority of sites that MethylToSNP identifies are meC > T SNPs, or neighboring sites affecting the probe. In these cases, C is the major allele and is consistently methylated. When replaced by a T allele, a false signal of differential methylation appears. By contrast, an unmethylated C major allele will give the same methylation value as a T allele. The reliability score $R$ represents a weighted measure based on the appearance of the data points for a given probe in the three *β*-value tiers, defined as “high” (> 0.75), “low” (< 0.25) and “middle” (between 0.25 and 0.75), with number of samples in each tier represented as $N_{\text{H}} , N_{\text{L}} , N_{\text{M}}$, respectively:

$$ R = \frac{1}{{N_{\text{H}} + N_{\text{M}} + N_{\text{L}} }}\left( {N_{\text{H}} + \frac{{N_{\text{M}} }}{2}} \right)\theta \left( {N_{\text{H}} } \right)\theta \left( {N_{\text{M}} } \right)\theta \left( {N_{\text{L}} } \right), $$

$$ \theta \left( n \right) = \left\{ {\begin{array}{*{20}c} {0:n = 0} \\ 1 \\ \end{array} } \right.. $$

If methylation values are falling in fewer than three tiers the reliability score of 0 is assigned.

We apply this stringent scoring approach to refine our datasets to those spanning the largest beta-value range, i.e., at the target CpG or the second position, as these locations have the greatest potential to impact the *p* values calculated for differential methylation between comparison groups.

To assess the reliability threshold necessary for calling SNP positions affecting the methylation interpretation, we calculated the scores for the simulated benchmark with two generated datasets (see Additional file 1). For the dataset with predetermined ratios of data points at each tier (which includes SNPs with low MAF) the mean reliability score was 0.568, whereas for SNPs with uniform distribution of methylation across tiers (corresponding to high MAF) mean reliability was 0.501 (Table 7). We assigned the threshold of 0.50 to reliability scores, with approximately 75% of all examples in the more realistic set-frequency dataset passing the threshold. When the data points are distributed mainly between the top two levels, this approach creates a theoretical reliability score of 0.75, whereas 0.50 is the expected value when all samples are evenly distributed across all three levels. Therefore, a higher reliability score represents a greater likelihood of the target site harboring an uncharacterized C to T SNP, consistent with a low-frequency T polymorphism being present and a higher concentration of samples falling within the top two tiers.

**Table 7:** Reliability scores from the simulated data sets

Data set	Mean reliability score	Median reliability score
Set frequency	0.568	0.553
Uniform frequency	0.501	0.500

### YRI HapMap dataset

We next tested MethylToSNP on data from YRI HapMap samples, some of which have both methylation and genotype data available. Methylation data were downloaded from Gene Expression Omnibus (GEO) project GSE26133 [16] for 77 samples and corresponding genotype data for available samples were found in the 1000 Genomes Browser (https://www.ncbi.nlm.nih.gov/variation/tools/1000genomes) [26]. One caveat with the browser data is that there were not genotype data at some methylation sites of interest for the samples which appeared polymorphic. For targeted sequencing, DNA samples were ordered from the Coriell depository and Sanger sequenced. The same samples were also subjected to targeted bisulfite sequencing to verify the methylation levels observed from the Illumina 450K methylation chip analysis.

### CEU HapMap dataset

Another group of well-studied samples, from individuals that likely have a very different epigenetic profile and genetic and life history from the individuals who contributed to the YRI (i.e., Yoruba in Ibadan, Nigeria) datasets, the CEU HapMap dataset, includes data from 90 Utah residents with Northern and Western European ancestry. Illumina 27K methylation data from the CEU sample set (from GEO project GSE27146 [17]) were subjected to MethylToSNP analysis.

### Southern African data analysis

To test MethylToSNP on primary samples, we used an in-house methylation dataset acquired from whole blood collected from peoples ethno-linguistically self-identifying as either KhoeSan or Bantu of Namibia, as in [27]. Few genomic data exist for these populations; less than ten genomes have been fully sequenced to date [21]. These populations harbor the greatest amount of genomic diversity, specifically the earliest diverged human lineage represented by people of KhoeSan ancestry [21], and population-specific SNPs are recorded in dbSNP. Nevertheless, many unidentified SNPs in this group may affect the interpretation of methylation studies—and MethylToSNP may detect them. Also, previously identified polymorphisms may not be present in the samples used in this study. The sample set contained 95 samples, 40 were KhoeSan, 51 were non-KhoeSan or Bantu-speaking southern Africans, and six were geographically matched Namibians of European descent, with two of the European controls run in duplicate for comparison. All samples were run on the Illumina 450K methylation chip (manuscript in preparation). The KhoeSan and control data were used to find sites that were differentially methylated between these two groups. This data set is broken down into three subsets for analysis: (i) all quality controlled methylation data from the chip (473,767 sites), (ii) all sites that are differentially methylated between the KhoeSan group and control group based on Mann–Whitney *U* tests (*p* ≤ 0.05) with Bonferroni test correction (*q* ≤ 0.05; 12,631 sites), (iii) the top 5% of differential methylation sites, ranked by largest magnitude of absolute difference, which are also statistically significant with Mann–Whitney *U* tests (*p* ≤ 0.05) and Bonferroni test correction (*q* ≤ 0.05), where known SNP positions are removed (400 sites).

### Regions of particular interest: CTCF sites and enhancers elements

We took an in-depth look at enhancer and CTCF sites implicated in differential methylation, where potential novel SNP content could confound methylation analysis. For example, a finding of differential methylation in a CTCF site could inhibit CTCF binding [28], as demonstrated at imprint control regions, such as *IGF2* and *H19*, where allele-specific methylation [29] inhibits binding. A SNP could also inhibit CTCF binding and present as differential methylation, impeding correct biological interpretation. Using the southern African dataset, we investigated how many differential methylation sites address these alternatives. The CTCF site locations were downloaded from the University of California, Santa Cruz Genome Browser [22, 30]. Likewise, sites of differential methylation that overlap known enhancer regions were intersected with our data to determine whether enhancer function could be impacted by the presence of SNPs or differential methylation. Enhancer site locations were downloaded with the Illumina 450K array annotation file and were originally compiled by Illumina from ENCODE projects. In order to maintain consistency of annotations in CTCF site analysis, we also downloaded a 450K array dataset (GEO GSE39672) for YRI and CEU HapMap samples.

## Supplementary information

**Additional file 1.** Supplemental Methods. Additional materials are provided for the determination of default thresholds (**Figure. S1**), assessment of false negative rates (**Figure. S2**), and inverse quantile weighting (**Figure. S3**).

## Data Availability

Data for YRI and CEU samples are publicly available. Data from KhoeSan sample data will not be publicly released due to sensitivity to KhoeSan data control requests. KhoeSan sequence or methylation data are available upon request to VMH.

## Abbreviations

SNP: single nucleotide polymorphism
CEU: Utah residents of European descent, from CEPH collection, HapMap
YRI: Yoruba, HapMap
EWAS: epigenome-wide association study
GEO: Gene Expression Omnibus

## References

[1] Chen YC, Sudre G, Sharp W, Donovan F, Chandrasekharappa SC, Hansen N, Elnitski L, Shaw P (2018). Neuroanatomic, epigenetic and genetic differences in monozygotic twins discordant for attention deficit hyperactivity disorder. Mol Psychiatry.

[2] Pubmed: The bibliographic database. https://www.ncbi.nlm.nih.gov/books/NBK153385/.

[3] Nardone S, Sams DS, Zito A, Reuveni E, Elliott E (2017). Dysregulation of cortical neuron DNA methylation profile in autism spectrum disorder. Cereb Cortex.

[4] Heyn H, Moran S, Hernando-Herraez I, Sayols S, Gomez A, Sandoval J, Monk D, Hata K, Marques-Bonet T, Wang L (2013). DNA methylation contributes to natural human variation. Genome Res.

[5] Daca-Roszak P, Pfeifer A, Zebracka-Gala J, Rusinek D, Szybinska A, Jarzab B, Witt M, Zietkiewicz E (2015). Impact of SNPs on methylation readouts by Illumina Infinium HumanMethylation450 BeadChip Array: implications for comparative population studies. BMC Genomics.

[6] Chen YA, Lemire M, Choufani S, Butcher DT, Grafodatskaya D, Zanke BW, Gallinger S, Hudson TJ, Weksberg R (2013). Discovery of cross-reactive probes and polymorphic CpGs in the Illumina Infinium HumanMethylation450 microarray. Epigenetics.

[7] Naeem H, Wong NC, Chatterton Z, Hong MK, Pedersen JS, Corcoran NM, Hovens CM, Macintyre G (2014). Reducing the risk of false discovery enabling identification of biologically significant genome-wide methylation status using the HumanMethylation450 array. BMC Genomics.

[8] Price ME, Cotton AM, Lam LL, Farre P, Emberly E, Brown CJ, Robinson WP, Kobor MS (2013). Additional annotation enhances potential for biologically-relevant analysis of the Illumina Infinium HumanMethylation450 BeadChip array. Epigenetics Chromatin.

[9] Zhou W, Laird PW, Shen H (2017). Comprehensive characterization, annotation and innovative use of Infinium DNA methylation BeadChip probes. Nucleic Acids Res.

[10] McCartney DL, Walker RM, Morris SW, McIntosh AM, Porteous DJ, Evans KL (2016). Identification of polymorphic and off-target probe binding sites on the Illumina Infinium MethylationEPIC BeadChip. Genom Data.

[11] Aryee MJ, Jaffe AE, Corrada-Bravo H, Ladd-Acosta C, Feinberg AP, Hansen KD, Irizarry RA (2014). Minfi: a flexible and comprehensive Bioconductor package for the analysis of Infinium DNA methylation microarrays. Bioinformatics.

[12] Fortin JP, Triche TJ, Hansen KD (2017). Preprocessing, normalization and integration of the Illumina HumanMethylationEPIC array with minfi. Bioinformatics.

[13] Pidsley R, Zotenko E, Peters TJ, Lawrence MG, Risbridger GP, Molloy P, Van Djik S, Muhlhausler B, Stirzaker C, Clark SJ (2016). Critical evaluation of the Illumina MethylationEPIC BeadChip microarray for whole-genome DNA methylation profiling. Genome Biol.

[14] Pidsley R, Wong CC, Volta M, Lunnon K, Mill J, Schalkwyk LC (2013). A data-driven approach to preprocessing Illumina 450K methylation array data. BMC Genomics.

[15] Andrews SV, Ladd-Acosta C, Feinberg AP, Hansen KD, Fallin MD (2016). “Gap hunting” to characterize clustered probe signals in Illumina methylation array data. Epigenetics Chromatin.

[16] Bell JT, Pai AA, Pickrell JK, Gaffney DJ, Pique-Regi R, Degner JF, Gilad Y, Pritchard JK (2011). DNA methylation patterns associate with genetic and gene expression variation in HapMap cell lines. Genome Biol.

[17] Fraser HB, Lam LL, Neumann SM, Kobor MS (2012). Population-specificity of human DNA methylation. Genome Biol.

[18] Sherry ST, Ward MH, Kholodov M, Baker J, Phan L, Smigielski EM, Sirotkin K (2001). dbSNP: the NCBI database of genetic variation. Nucleic Acids Res.

[19] Skakkebaek A, Nielsen MM, Trolle C, Vang S, Hornshoj H, Hedegaard J, Wallentin M, Bojesen A, Hertz JM, Fedder J (2018). DNA hypermethylation and differential gene expression associated with Klinefelter syndrome. Sci Rep.

[20] Abi Habib W, Brioude F, Azzi S, Rossignol S, Linglart A, Sobrier ML, Giabicani E, Steunou V, Harbison MD, Le Bouc Y (2019). Transcriptional profiling at the DLK1/MEG3 domain explains clinical overlap between imprinting disorders. Sci Adv.

[21] Schuster SC, Miller W, Ratan A, Tomsho LP, Giardine B, Kasson LR, Harris RS, Petersen DC, Zhao F, Qi J (2010). Complete Khoisan and Bantu genomes from southern Africa. Nature.

[22] Kent WJ, Sugnet CW, Furey TS, Roskin KM, Pringle TH, Zahler AM, Haussler D (2002). The human genome browser at UCSC. Genome Res.

[23] Jain M, Koren S, Miga KH, Quick J, Rand AC, Sasani TA, Tyson JR, Beggs AD, Dilthey AT, Fiddes IT (2018). Nanopore sequencing and assembly of a human genome with ultra-long reads. Nat Biotechnol.

[24] R Core Team (2016). R: a language and environment for statistical computing.

[25] Wang H, Song M (2011). Optimal *k*-means clustering in one dimension by dynamic programming. R J.

[26] Auton A, Brooks LD, Durbin RM, Garrison EP, Kang HM, Korbel JO, Marchini JL, McCarthy S, McVean GA, The 1000 Genomes Project Consortium (2015). A global reference for human genetic variation. Nature.

[27] Petersen DC, Libiger O, Tindall EA, Hardie RA, Hannick LI, Glashoff RH, Mukerji M, Fernandez P, Haacke W, Indian Genome Variation C (2013). Complex patterns of genomic admixture within southern Africa. PLoS Genet.

[28] Wang H, Maurano MT, Qu H, Varley KE, Gertz J, Pauli F, Lee K, Canfield T, Weaver M, Sandstrom R (2012). Widespread plasticity in CTCF occupancy linked to DNA methylation. Genome Res.

[29] Szabo PE, Tang SH, Silva FJ, Tsark WM, Mann JR (2004). Role of CTCF binding sites in the Igf2/H19 imprinting control region. Mol Cell Biol.

[30] Karolchik D, Hinrichs AS, Furey TS, Roskin KM, Sugnet CW, Haussler D, Kent WJ (2004). The UCSC Table Browser data retrieval tool. Nucleic Acids Res.

