# Supplementary material for: MethylToSNP: identifying SNPs in Illumina DNA methylation array data
Source: Epigenetics Chromatin. 2019 Dec 20;12:79. doi: 10.1186/s13072-019-0321-6 (PMC6923858; doi:10.1186/s13072-019-0321-6)
Supplement: Supplementary file 1 — Additional file 1. Supplemental Methods. Additional materials are provided for the determination of default thresholds (Figure. S1), assessment of false negative rates (Figure. S2), and inverse quantile weighting (Figure. S3). [file 13072_2019_321_MOESM1_ESM.docx]

### **1. Threshold testing for gap_ratio and gap_sum_ratio values on large simulated data sets**

In order to test the performance of SNP detection we simulated two datasets, 95 samples each, and assessed a range of threshold parameter values for MethylToSNP algorithm. In the first dataset, methylation values were placed into one of the three tiers (85-100%, 40-60% or up to 15%) to meet pre-chosen allele frequencies (see Additional file 1, Table S1) incorporating biases to reflect the presence of rare alleles, known as the “set-frequency” dataset. In the second dataset, the counts of simulated SNP probes were uniformly placed into the three methylation tiers, known as the “uniform frequency” set, with 32 samples in each of the three tiers, thus representing SNPs with MAF close to 0.5. In each dataset, half of the probes had the SNP-like methylation pattern (i.e., producing the three tiers of methylation β-values) and the other half of probes was sampled from real array measurements. In the set-frequency data matrix, 4,898 of 10,000 simulated probes are SNP positions. In the uniform-frequency matrix 4,961 of 10,000 simulated probes are SNP positions.

**Additional file 1, Table S1. Probabilities for generating set-frequency SNP dataset with 95 samples.**

| Probability | Number of samples with beta 0.85-0.1 | Number of samples with beta 0.4-0.6 | Number of samples with beta 0-0.15 |
| --- | --- | --- | --- |
| 30% | 45 | 40 | 10 |
| 20% | 5 | 10 | 80 |
| 30% | 30 | 25 | 30 |
| 20% | 85 | 7 | 3 |

To assess these datasets, we determined probes with values matching the expected 3-tiered pattern of SNP positions using the MethylToSNP algorithm without outlier removal. We assessed the performance of detection of simulated SNP probes at varying parameter combinations. For instance, the *gap-sum* parameter was varied in increments of 0.05 in a range from 0.35 to 0.65, while holding the *gap* parameter at 0.75. The number of SNP predictions at each increment are plotted in Additional file 1 Figure S1A.


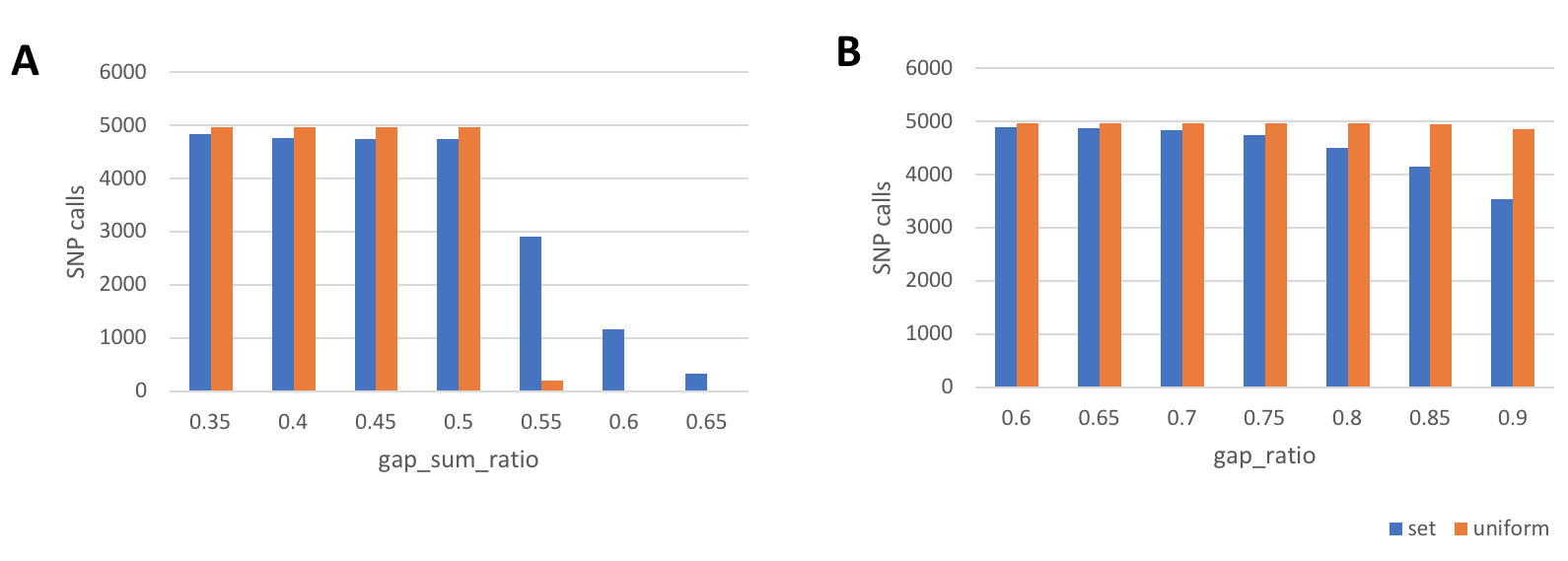


Additional file 1, Figure S1. (A) Number of SNP calls when altering the *gap_sum_ratio* parameter for simulated datasets (100 loci and circa 10,000 data points) representing different MAF values: set frequency for low MAF and uniform frequency for high MAF. (B) Numbers of SNP calls detected in two simulated data sets while varying the *gap_ratio* threshold. The maximum recovery for the uniform frequency dataset is 4961 and for the set frequency dataset is 4898. No false positives were predicted

Increasing the *gap_sum_ratio* threshold shows how SNP prediction outcomes change when more whitespace is present in the range of beta values. For example, predictions are best for the uniform-frequency dataset (orange bars), because the data points are evenly represented across the three tiers, enabling gap finding to work well. In the range of *gap_sum_ratio* parameter between 0.35 and 0.50, SNP predictions perform consistently well, but fail above 0.50 when two largest gaps are required to cover more than 50% in the data spread).

SNP predictions do not perform as well on the dataset generated to have a set-frequency data distribution (i.e., blue bars), where the data points are not evenly distributed across the three methylation value tiers, as might be the case for rare SNP alleles.

We repeated the same analysis by varying the *gap_ratio* threshold in increments of 0.05 in a range from 0.60 to 0.90, while holding the *gap_sum_ratio* value constant at 0.5 (Additional file 1, Figure S2). Here, SNP recall is 100% in the uniform-frequency dataset (orange bars), except at the 0.9 level, when both gaps are required to be almost exactly the same. Below *gap_sum_ratio* value of 0.50, performance remains consistently high.

Altering the *gap_ratio* threshold shows that SNPs with rare allele frequencies (set-frequency dataset) are more difficult to detect (i.e., blue bars). The performance remains relatively consistent over a range of *gap_ratio* thresholds.

***2.* Assessment of false negative discovery rate depending on *gap_ratio* and *gap_sum_ratio* parameters on true SNPs**


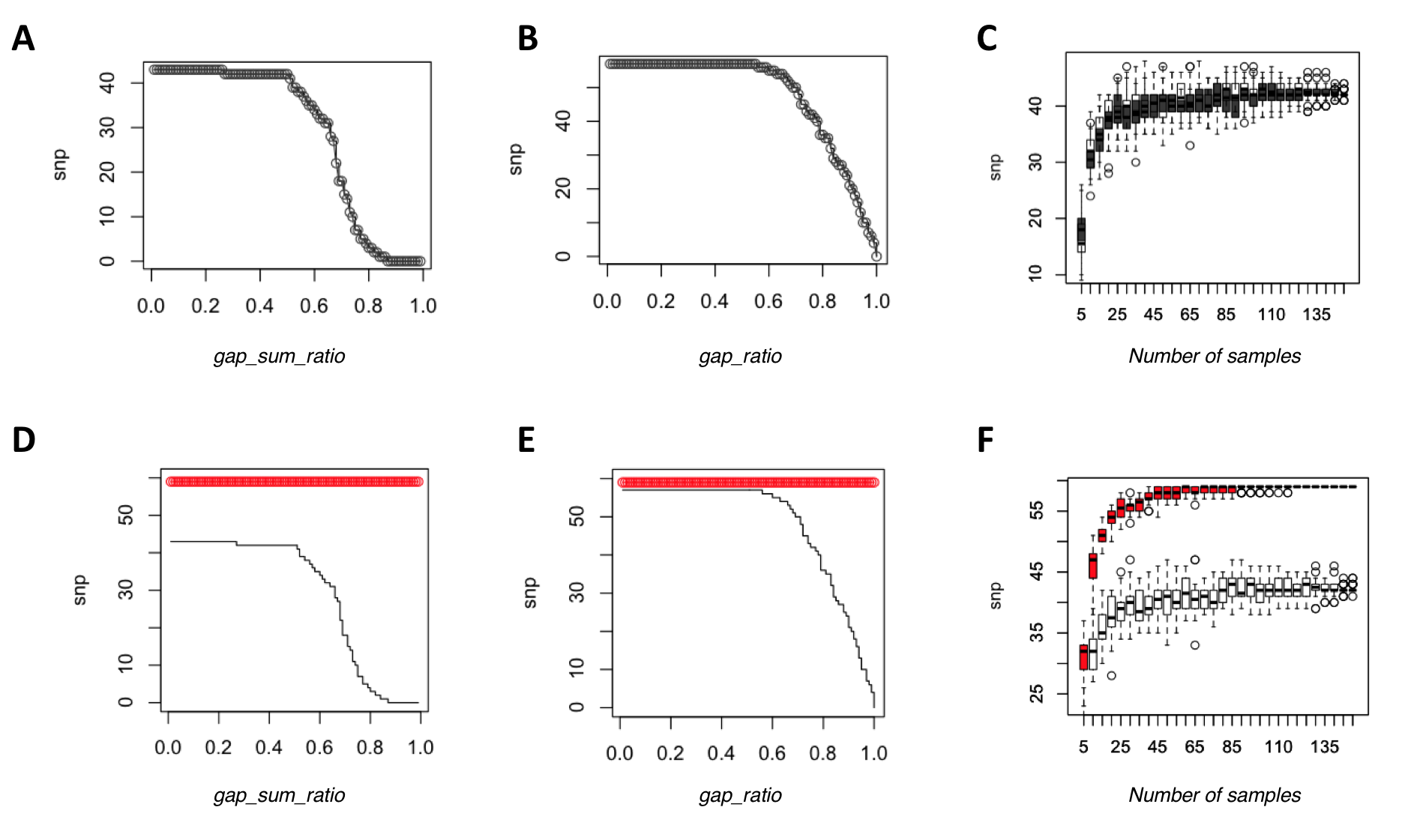


Additional file 1, Figure S2. Numbers of SNP calls detected in “true-SNP” data set with 59 probes and 152 samples while varying the (A, D) *gap_sum_ratio* threshold, while *gap_ratio*=0.75 or (B,D) *gap_ratio* threshold while *gap_sum_ratio*=0.5 or (C, E) subsampling 100 times without replacement smaller data sets with less patient samples than in the original dataset. The plots in the first row (A, B, C) shows results for MethylToSNP run without outlier removal (same as in Additional file 1, Figure S1); the second row (D, E, F) shows results for MethylToSNP with outlier removal at *outlier_sd*=3 threshold in red color and for comparison shows the same data as in the row above in black color

The second benchmark uses a very conservative definition of a true SNP that have been intentionally added by array designers as control probes. Illumina EPIC array includes 59 of such “rs” probes. We used the unnormalized methylation values of these “true SNP” probes in 152 pediatric samples from GEO dataset GSE137682. Additional file 1, Figure S2 shows the results of the benchmark depending on the MethylToSNP parameters. The curves show response similar to Additional file 1, Figure S1. The benchmark shows that although the default parameters could not achieve a complete recall, they are a safe conservative choice for detection of three-tier patterns in methylation data. Outlier detection affects calculation of gaps between clusters and allows to detect three-tier patterns even in the presence of noise and other confounding factors. Additional file 1, Figure S2D,E shows performance of outlier removal option in red color.

Similarly, the benchmark measures the effect of a small dataset on the false negative rate, where there is insufficient data to detect a three-tier pattern. The benchmark shows (Additional file 1, Figure S2C,F) that for high minor allele frequency SNPs (MAF ~ 0.5) the recommended number of samples is at least 50. In case of rare alleles, the data samples carrying the rare alleles will need to be part of the dataset, otherwise, obviously, they could not be detected.


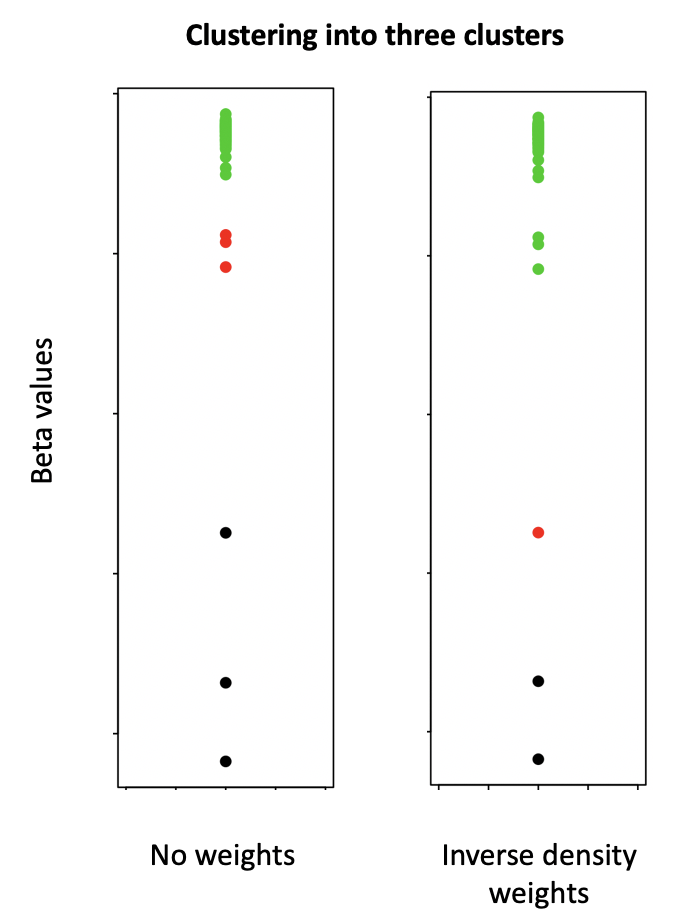


Additional file 1, Figure S3. Comparison of clustering of beta values with and without inverse quantile weighting illustrated on the YRI beta-values at cg21226234 probe. Clustering arrangement for three clusters (high, middle, low) is shown by color (green, red, black, respectively)
